# A Case of Subacute Cutaneous Lupus Erythematosus in a Patient with Mixed Connective Tissue Disease: Successful Treatment with Plasmapheresis and Rituximab

**DOI:** 10.1155/2013/857694

**Published:** 2013-07-28

**Authors:** M. Fantò, S. Salemi, F. Socciarelli, A. Bartolazzi, G. A. Natale, I. Casorelli, A. Pavan, S. Vaglio, R. Di Rosa, R. D'Amelio

**Affiliations:** ^1^Department of Allergy, Clinical Immunology and Rheumatology, S. Andrea Hospital, Sapienza University of Rome, Italy; ^2^Department of Pathology, S. Andrea Hospital, Sapienza University of Rome, Italy; ^3^Department of Immunohematology and Transfusion Unit, S. Andrea Hospital, Sapienza University of Rome, Italy

## Abstract

A 30-year-old woman affected by Mixed Connective Tissue Disease with scleroderma spectrum developed a facial eruption, a clinical and histological characteristic of subacute cutaneous lupus erythematosus (SCLE). Speckled anti-nuclear antibodies, high-titer anti-ribonucleoprotein1, anti-Sm, anti-Cardiolipin (aCL) IgG/IgM, and anti-Ro/SSA antibodies were positive. SCLE was resistant to Azathioprine, Hydroxychloroquine, and Methotrexate while Mycophenolate Mofetil was suspended due to side effects. Subsequently, the patient was treated with three cycles of therapeutic plasma exchange (TPE) followed, one month after the last TPE, by the anti-CD20 antibody Rituximab (RTX) (375 mg/m^2^ weekly for 4 weeks). Eight and 16 months later the patient received other two TPE and RTX cycles, respectively. This therapeutic approach has allowed to obtain a complete skin healing persistent even after 8-month follow-up. Moreover, mitigation of Raynaud's phenomenon, resolution of alopecia, and a decline of aCL IgG/IgM and anti-Ro/SSA antibodies were observed.

## 1. Introduction

 Mixed Connective Tissue Disease (MCTD) is currently defined as an overlapping syndrome with clinical features of Systemic Sclerosis (SSc), Systemic Lupus Erythematosus (SLE), Rheumatoid arthritis (RA), and Polymyositis/Dermatomyositis (PM/DM); the presence of high-titer anti-ribonucleoprotein1 (U1RNP) or speckled anti-nuclear antibodies (ANA) at titer ≥1 : 2,000 is necessary for the diagnosis. The disease affects mainly women in the third decade of life (from 80 to 90%) but it has been also reported in children and in over-80-year-old people [1].

The most frequent clinical manifestations are Raynaud's phenomenon (RP), swollen hands, sclerodactyly, arthritis, myalgias, and oesophageal dysmotility, and also alopecia, malar rash, lymphadenopathy, or kidney damage can be present. Rarely, subacute cutaneous lupus erythematosus (SCLE), characterized by annular or papulosquamous lesions, photosensitivity, and presence of anti-Ro/SSA and anti-La/SSB antibodies, has been described in MCTD patients [2, 3]. MCTD therapy should be identified for each patient depending on the affected organ, but generally there is a good response to steroids, different types of vasodilators, and immunosuppressive agents such as Hydroxychloroquine (HCQ), Azathioprine (AZA), Methotrexate (MTX), or Cyclophosphamide (CYC) [1].

## 2. Case Presentation

**Figure 1:**
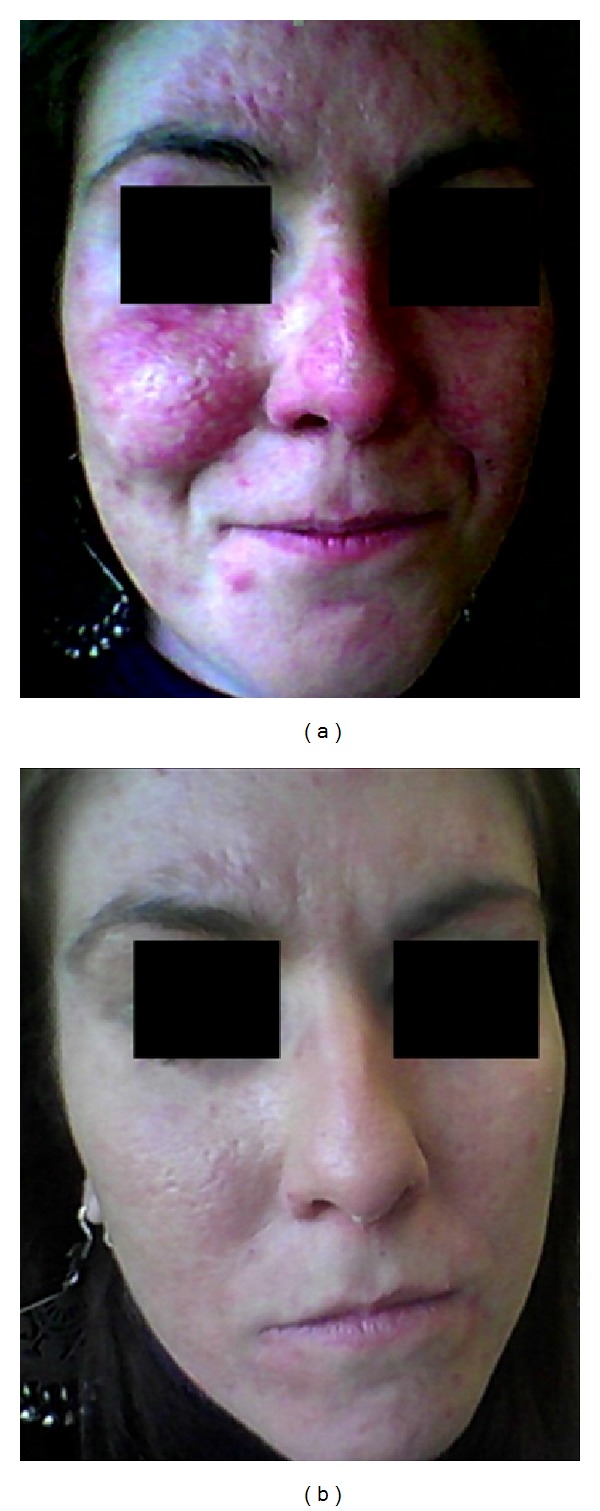
(a) SCLE: cutaneous eruption which infiltrates forehead, cheeks, and chin. (b) Disappearance of facial SCLE after the third cycle of TPE plus RTX.

**Figure 2:**
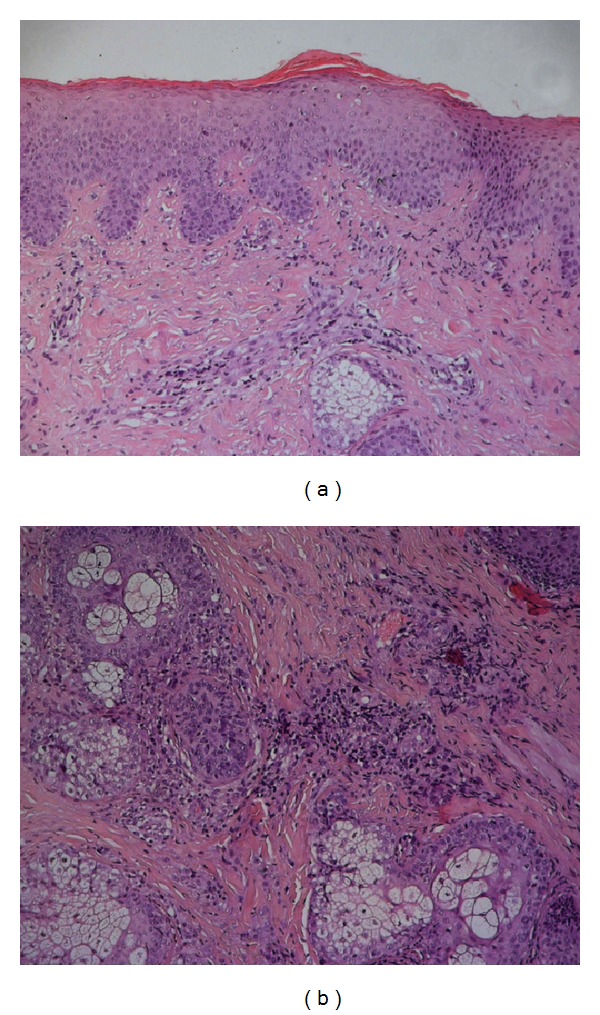
(a) The epidermal layer is characterized by a mild degree of papillomatosis, acanthosis, and focal mixed orthokeratotic and parakeratotic hyperkeratoses. The underlying papillary and reticular dermis shows a marked fibrotic change associated with a chronic mononuclear perivascular inflammatory infiltrate (H&E, 100x magnification). (b) A moderate mononuclear chronic infiltrate is present around adnexal structures (H&E, 100x magnification).

A case of a thirty-year-old woman affected by MCTD with scleroderma spectrum and epilepsy since she was fifteen is here reported. At the beginning she presented fever up to 40°C, arthalgias mainly at knees, wrists, and shoulders, and increased levels of erythrocyte sedimentation rate (ESR) and C-reactive protein (CRP). She also had speckled type of ANA up to 1 : 2,560, anti-U1RNP, anti-Sm, anti-Cardiolipin (aCl) IgG and IgM positivity, hypergammaglobulinemia, myositis, lymphopenia, RP, cutaneous calcinosis, and scleroderma. She started treatment with Cyclosporine A (CYA), corticosteroids (CCS), and nifedipine in 1998. The following year myositis worsened with an increase of Creatinphosphokinase (CPK) up to 8,000; thus she received pulse steroid therapy, 800 mg/die methylprednisolone, monthly for six months; four years later, in 2001, because of exacerbation of arthralgias she started HCQ, with satisfying improvement. In August 2003 a grade C esophagitis and a diffuse bilateral interstitial lung disease with severe decrease of carbon monoxide diffusing capacity (DLCO) were detected. Irregular urticarial lesions in her arms and chest and purpura in her legs and alopecia also arose. Thus she started AZA, with lung and cutaneous improvement. In 2007 CYA was suspended after a blood pressure increase. Subsequently, a facial eruption appeared in correspondence of forehead, cheeks, and chin (Figure 1(a)). Histopathological examination of a skin biopsy revealed “a skin characterized by modest papillomatosis, acanthosis, and focal hyperkeratosis of the epidermis. The superficial and deep dermis showed marked sclerosis associated with lymphomononuclear perivascular and periadnexal cellular infiltrate” (Figures 2(a)-2(b)).

Direct immunofluorescence on frozen skin biopsy (“lupus band test”) demonstrated “a dust-like IgG particles staining pattern consisting of fine granular Ig deposition scattered through the epidermis.” This picture has been reported to be specific for SCLE [4]. Taking into account the clinical and histological features and anti/Ro antibodies positivity, a diagnosis of subacute cutaneous lupus erythematosus (SCLE) associated to dermal sclerosis was made. In 2008 MTX and the subsequent year Mycophenolate Mofetil (MMF) were able to induce a slight skin improvement but they were stopped due to inefficacy and excessive weight loss, respectively. Therefore, in December 2010, therapeutic plasma exchange (TPE) was performed, every other day for a total of five exchanges, using albumin to replace the plasma removed. The same cycle was repeated in February and in March 2011 followed, one month after, by Rituximab (RTX) (375 mg/m^2^ weekly for 4 weeks). Eight and 16 months later the patient received other two TPE procedures followed by RTX (375 mg/m^2^ weekly for 4 weeks), respectively. After the first 3 TPE cycles there was a slight improvement of SCLE and the addition of RTX has allowed for obtaining a further clearing of facial eruption, while 8 weeks after the last RTX infusion a complete skin healing was reached and is still persistent after 8-month follow-up (Figure 1(b)). Mitigation of RP and resolution of alopecia were also observed. During this period no flares-up were observed and the patient assumed only HCQ and a low-dose of CCS (5 mg/die). Interestingly, aCl-IgG/IgM and anti-Ro/SSA antibodies disappeared after the first TPE and RTX treatment, whereas they showed a slight increase before the third cycle at the end of which they became definitively negative for the next 8 months.

## 3. Discussion 

Successful off-label use of RTX in SLE manifestations as cytopenia, diffuse erythematosus lesions, and alopecia or as rescue therapy in life-threatening complications of several autoimmune diseases has been sometimes reported [5, 6]; in addition, the anti-CD20 therapy has even been employed in dermatologic field including blistering diseases, graft versus host disease, and DM/PM but, to our knowledge, only one case of refractory SCLE treated with RTX has been described [7]. Recently, the role of B cells in the pathogenesis of SSc has been underlined, and the RTX efficacy to improve skin fibrosis and pulmonary function has been reported [8, 9].

Regarding MCTD, a successful treatment with RTX (in combination with CCS, CYC, and iloprost) has been reported in a case of severe, refractory RP [10], while on the contrary, Dunkley et al. reported a case of MCTD with scleroderma spectrum in which RTX was not able to control RP [11]. Moreover two MCTD cases in which TPE was able to treat visceral RP with multiple organ damage (treated with combination with CYA and CCS) [12] and acute renal failure (in combination with CYC and captopril) [13] have been described.

Plasmapheresis, in association with RTX, has been used in only one case of MCTD patient, in whom were observed RP resolution, ANA, anti-centromere (CENP-B) antibodies, and decrease of serum IgG-IgM [14].

A case of MCTD patient with scleroderma spectrum, in whom therapy-resistant facial SCLE was completely resolved after combination of TPE and RTX (375 mg/m^2^ weekly for 4 weeks), is here reported. Moreover, we observed RP improvement and alopecia, aCl, and anti-Ro/SSA serum antibodies disappearance. Anti-Ro/SSA antibodies are closely associated with SCLE, and the resolution of clinical features accompanied by the disappearance of these antibodies from serum strongly suggests a primary role of anti-Ro/SSA autoantibodies in the pathogenesis of SCLE.

Before starting this new approach, in order to exclude a iatrogenic cause in SCLE induction, carbamazepine and nifedipine were replaced with similar noninducing drugs, but no clinical effects on SCLE were observed [15].

In conclusion, association of TPE and RTX should be considered as a valid and safe therapeutic tool for controlling SCLE in therapy-resistant MCTD. Moreover, despite the relative short follow-up, the intriguing observation of the beneficial effects that this treatment could exert also on cutaneous sclerosis occurring in MCTD patients makes this therapeutic approach very promising.
